# Tuberculosis of the knee: A pitfalls in clinical settings (A case report and literature review)

**DOI:** 10.1016/j.ijscr.2020.04.090

**Published:** 2020-05-12

**Authors:** Noni Novisari Soeroso, Fannie Rizki Ananda, Heru Rahmadhany, Dedy Dwi Putra

**Affiliations:** a Department of Pulmonology and Respiratory Medicine, Faculty of Medicine, Universitas Sumatera Utara, Universitas Sumatera Utara Hospital, Jl. Dr Mansyur No. 66, Medan 20154, Indonesia; b Department of Orthopaedic and Traumatology, Faculty of Medicine, Universitas Sumatera Utara, Universitas Sumatera Utara Hospital, Jl. Dr Mansyur No. 66, Medan 20154, Indonesia; c Department of Radiology, Faculty of Medicine, Universitas Sumatera Utara, Universitas Sumatera Utara Hospital, Jl. Dr Mansyur No. 66, Medan 20154, Indonesia

**Keywords:** Knee, Tuberculosis, Rare case, Surgery

## Abstract

**Introduction:**

Tuberculosis of the knee is a rare form of diseases associated with tuberculosis that is frequently misdiagnosed as malignancy or bacterial infections. Its symptomatology might be identical to other conditions; thus finally turns the clinician misdiagnosed of the patient circumstances.

**Presentation of the case:**

A 31-year-old male patient presented a unilateral knee swelling, reddish, warmth, pain, with a limited range of movement and then diagnosed with tuberculosis of the knee, histopathologically confirmed following surgical removal, including synovectomy without arthroplasty procedure. Furthermore, the patients underwent the administration of an anti-tuberculous drug regiment for a 1-year period. There was clinical improvement attained afterward after the prompt management since the initiation of therapy progressively.

**Discussion:**

This case is unique due to the unspecific clinical manifestations of the disease, which contributed to delay diagnosis. Further, either the patients and his parents still had a lack of knowledge according to his condition. Opposite to the recent studies, there was an excellent outcome after simple surgical procedure, including synovectomy without arthroplasty followed by 1 year of anti-tuberculous drug.

**Conclusion:**

Knee tuberculosis is a rare disease that often misdiagnosed as bacterial infections or malignancy. Mimicking clinical manifestations needs further surgical approach followed by histopathology examination to diagnose this disease. Early recognition and prompt treatment were crucially required to avoid the permanent limitation of movement that could affect the patient’s quality of life. In this case, a lack of knowledge of the patient and his family significantly contributes to the delayed diagnosis.

## Highlights

• We herein present a case of an unusual manifestation of knee tuberculosis. Its slowly progressive disease characteristics after being ignored for 5 years interest the authors to report this case report. Without doing arthoplasty, synovectomy followed by 1 year of anti-tuberculosis treatment give positive improvement of the patient where he can continue his job and daily activities as before he got the disease 5 years ago. Slight deformity resulted in muscle stiffness may be restorated after few months of physical rehabilitation.
• Further monitoring is needed anually for assessing the long term complications of knee tuberculosis, including early secondary osteoarthritis.
• The place of conducting the case report in Indonesia, mainly in Sumatera Utara. Thus, we hope that the manuscript fits the scope of International journal for Surgery Case Report. The article is original, unpublished, and not being considered for publication elsewhere.

## 1 Introduction

Tuberculosis (TB) has been recognized as a disease with many mimicry clinical manifestations that often lead to being underdiagnosed [1]. According to the World Health Organization (WHO) in 2019, Indonesia is the third country with the highest prevalence of tuberculosis. Indonesia was also included in 10 states that have the most substantial gap between the number of new cases reported and the 10 million incident cases in 2018 [2]. In China’s population, extra-pulmonary TB was accounted for more than a third of all TB cases, with the highest incidence was skeletal and pleural manifestations [3]. Skeletal TB manifestation itself contributed almost a third of the total extra-pulmonary signs with the highest rate occurred in the spine, hip, and knee [4]. Mimicry manifestation of this disease provides difficulties in diagnosing it, particularly in extra-pulmonary TB [5]. Subsequently, this disease is also often manifested as acute infection and malignancy, so that delayed or underdiagnosed and medical treatments often occur.

In this study, we reported an immune-competent 31-year-old man with unilateral knee swelling, warmth, and pain that has been underdiagnosed for 5 years. This study had been written according to SCARE 2018 guidelines for case report [6].

## 2 Case reports

A 31-year-old man, was admitted to the outpatient clinic with major complaints of reddish skin transformation, warmth, and swelling of the right knee that progressively occurred since 2013; there was also intermittent pain that has been experienced since the symptoms emerged. The pain characteristically was exacerbated after strenuous activities without respiratory manifestations were found. Losing appetite and weight for 3 kg in one month has been suffered, and no symptoms of night-sweats and chills are experienced. Contact history with TB patients was not found, but a traumatic injury occurred in September 2012. The patient had a motorcycle accident, injuring the same leg. The history of medication was analgesics and oral antibiotics, including cephalosporin. There were no allergic indications, including food and drugs. There was no history of malignancy or genetic abnormality in his family background.

In 2013, this patient was admitted to another hospital and had been diagnosed with acute infections with malignancy as a differential diagnosis. The patient was suggested to undergo further diagnostic examinations. However, he preferred to undergone alternative treatments such as massage and herbal medicines. The patient and his parents still thought that his condition was not dangerous to get surgery. After several years of these treatments, no improvement of symptoms and clinical conditions were reported. The swelling of the leg became more severe, with the increased frequency of pain exacerbated after 5 years of alternative medicine approach.

Physical examinations have reported the presence of swelling, reddish, and warm through palpation of the right knee with impaired proper motions. The affected extremity could not be flexed under 110 °C, and extended −30° with no abnormality was found based on physical examinations of the spine, hip, and left knee. Meanwhile, chest radiograph demonstrated infiltration in both upper lobe lungs.

**Fig. 1:**
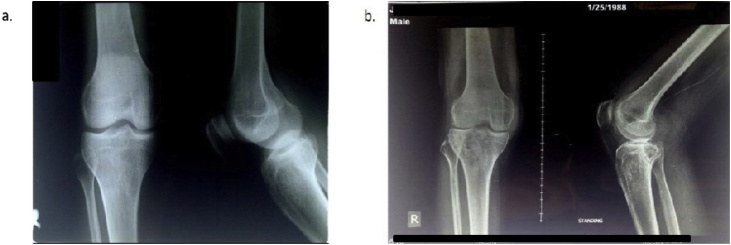
Knee Radiograph. (a) In 19 November 2013, it is shown the narrowing of knee joint gap. (b) In 18 January 2016, it can be observed that there has been a lytic lesion with ill-defined in periarticular projection in right proximal tibia and there was an ill-defined focal thickening in suprapatella and posterior of the right knee suspected a primer bone lesion in peri-articular proximal tibia

From the sequence radiograph examinations, it showed the progression of knee destruction. In 2013, there was only a narrowing of the joint gap (Fig. 1a). Still, after being ignored for 3 years, it has progressed to the bone destruction, which was indicated by the presence of a lytic lesion in both fibula and tibia (Fig. 1b).

**Fig. 2:**
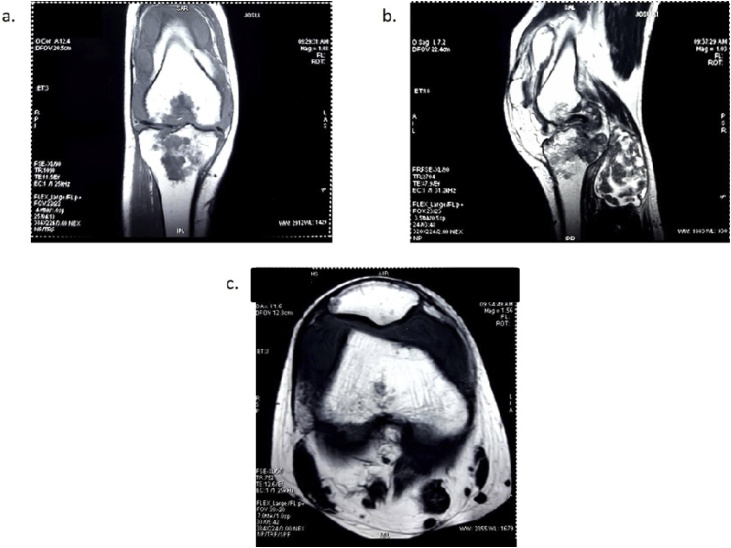
MRI examination of the knee. (a) Based on coronal view, it showed well-defined iso-hyperintensed lesion and fat-set epimetaphysis femur (distal); tibia-fibula epimetaphysis (proximal); half of right inferior patella pole. (b) In sagittal T2 posterior projection of right genu, there was an iso-hyperintense mass that involved half of musculus gastrocnemius (mediolateral), plantaris, popliteal, and half of femoral arterial and venous and tibial nervesagittal view. (c) From the axial view, there was a free fluid collection in which dominant in suprapatella and minimal infrapatella projection.

**Fig. 3:**
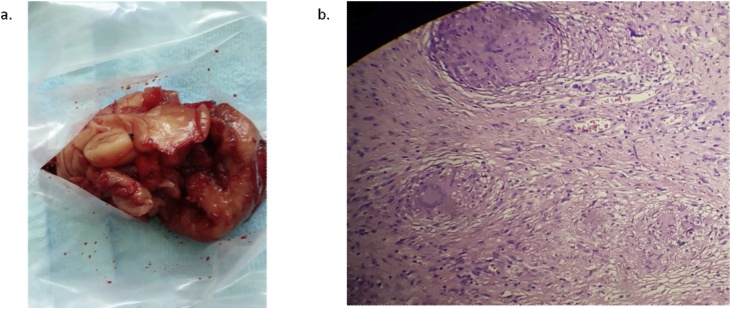
The macroscopic and microscopic appearance of tuberculosis of the knee. (a) The macroscopic examination showed the isolated of synovium destruction with cold abscess. (b) The microscopic examination revealed epitheloid proliferation, granuloma, necrotic, lymphocyte infiltration, and localized fibrosis, suspect of chronic specific inflammation including tuberculosis mass.

Finally, another modality which has favorable of soft tissue infiltration, Magnetic Resonance Imaging (MRI) was used later. It showed the well-defined iso-hyperintense mass and free fluid collection that lead to right tuberculous knee arthritis with posterior abscess and synovitis infection (Fig. 2). Based on blood examination, no abnormality of complete blood count and thrombosis parameters were found. The patient was performed synovectomy in June 2018 by an orthopedist. Spinal anesthesia was performed before debridement and arthrotomy, followed by total synovectomy. The medial incision of the right patella revealed the synovial mass without any attachment to the adjacent structure (Fig. 3a). Later, histopathology revealed the epitheloid proliferation, granuloma, necrotic, lymphocyte infiltration, and localized fibrosis, suspecting a chronic specific inflammation, which included tuberculosis mass (Fig. 3b). Then, the patient was diagnosed with knee tuberculosis.

**Fig. 4:**
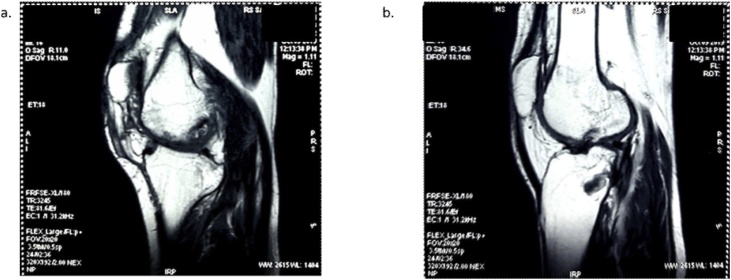
In coronal and sagittal view showed the prominent lytic lesion in femur, tibia-fibula bone.

Patient was prescribed with isoniazid (10 mg/kg/day), pyrazinamide (30 mg/kg/day), rifampicin (15 mg/kg/day), ethambutol (15 mg/kg/day), and streptomycin (25 mg/kg/day) daily for 2 months followed the synovectomy procedure. Treatment was then continued for 9 months with isoniazid (10 mg/kg/day) and rifampicin (15 mg/kg/day). Clinical improvement was observed after eight months of treatments; it was clinically proven that the improvements on flexed and extended ability to 130° and −10°. The side effect of treatments did not really affect the patient’s condition. After 1 year, the patient has shown no clinical symptoms with the exception of limited flexibility movement (maximal flexed ability 160°), and there was an improvement of radiologic examinations. After a year, follow-up x-ray examinations on the chest showed scanty fibrotic in the upper pulmonary lobe. MRI was performed, and it showed the more prominent of the lytic lesion compared with the previous MRI without secondary infection (Fig. 4). The patient was suggested to attend physical rehabilitation to ameliorate his joint movement without arthroplasty procedure scheduled. This time, he has been work as before he suffered the disease without any serious limitation. Clinical and radiological followed up might be considered to detect the long term complication, including secondary osteoarthritis.

## 3 Discussion

Tuberculosis has been considered as significant health problems, infecting almost a quarter of the population around the world, with the highest incidence in productive age [2]. Tuberculosis can manifest in the condition of extra-pulmonary, including lymph node (the most common), skin [7], meninges, and bone [8]. In our case report, the patient was 31-year-old Indonesian with a diagnosis of tuberculosis of the knee.

From the clinical findings, almost all the patients have painful knee with the presence of abscess (16%) and sinus (42%) [5]. In this case, our patients presented a progressive swollen and reddish knee with intermittent pain, and this patient also had a posterior abscess in his knee as a secondary infection after tuberculosis. In tuberculosis of the knee, the edema was monoarticular, slowly progressive, with slow and gradual pain. As usual, the patient realized the symptoms because of the limitation of movement that is felt after a higher degree of joint destructions [9]. In this case, as the pain was intermittent, patients have experienced slight disturbances in his clinical manifestations until the edema limited his movement to do his regular activities and forced him to resign from his job.

History of trauma sometimes is considered as a confounding factor to diagnose the tuberculosis of the knee [9]. In some literature, the history of twisted joint or strain is often preceding the development of symptoms [9]. In this case, the patient also has a history of a motorcycle accident on the same leg that could lead to a diagnosis of acute infections in the knee.

In this case, we have the serial of Knee X-Ray photographs in which in 2013, there was a narrowing of the joint gap without the structural destruction in bone. However, after being neglected for 3 years, it developed into a bone lesion in peri-articular proximal tibia based on Knee X-Ray in 2016. From the Magnetic Resonance Imaging in 2018, it suggested that chronic tuberculous knee arthritis with posterior abscess and synovitis infection occurred. In recent literature, the radiological findings of the tuberculosis of the knee are destructions of the knee joint with a lytic lesion, depending on the degree of damage. Despite the Phemister triad, the radiological findings of tuberculosis of the knee are in juxta-articular osteopenia, joint space narrowing, and erosions. In this case, the patient has all of these categories, so the diagnosis has been pursed into tuberculous arthritis with secondary infections. Based on a study conducted by Kerri and Martini [10], tuberculosis of the knee is divided into four stages in term of its radiographic observation

In this case, the patient was categorized in Stage 2 with one or more erosions (cavities) on the bone. According to ISTC 3, histopathology examination was critical in diagnosing extra-pulmonary tuberculosis [11], which would show a central necrotic area with the infiltration of lymphocytes, histiocytes, a Langhan’s giant cell, mantle cell, and fibrous tissue. In addition, there was a central necrotic area surrounded by histiocytes and a few giant cells that have nuclei marginally [12,13].

Invasive treatments, including surgical removal of the involved joint, which is followed by post-operative anti-tuberculous therapy, provide positive outcomes in patients with knee tuberculosis [[14], [15], [16]]. Total knee arthroplasty (TKA) is considered as the primary treatment of knee tuberculosis with a lower rate of recurrence as well as anti-tuberculous chemotherapy for 11 years follow-up [17]. The consensus about the surgical timing, prosthesis selection, and the timing for starting anti-tubercular therapy has not been established yet^16^]. In this case, TKA was not performed, but a synovectomy is a surgical intervention, a significant clinical improvement without the need for arthroplasty was found after 6 months of follow up. This is in accordance with a study conducted by Misgar that involved 50 patients with TB genu undergoing synovectomy procedure as well as Shen, who included ten patients. Both of these studies showed that synovectomy with post-operation anti-tubercular therapy had a good outcome in the early stage of synovial tuberculosis of the knee joint [18,19]. Recently, arthroscopic management is considered as an alternative treatment in the early stage of knee tuberculosis, but this treatment could not be performed due to lack of facilities.

A regiment of anti-tubercular therapy differs among several studies. In this study, a synovectomy with rifampicin (R), isoniazid (H), ethambutol (E), pyrazinamide (Z), and streptomycin (S) for 2 months following by R and H for nine months have shown improvements in symptoms and radiologic features. Similarly, Uboldi et al. have performed the poly-therapies of R, Z, and E for 1 year, and the therapies have also reported improvements in the symptoms. However, according to the guideline of tuberculosis, standard TB treatments of extra-pulmonary tuberculosis are divided into two phases; the intensive phase with the combination of R, H, Z, E, and the continued phase includes R and H [5,20] for more than 1 year.

After 1 year of follow up, the patient has displayed no clinical symptoms with the exception of slight limitations of flexed movements. This might be the result of muscle stiffening or the destruction of the knee joint. Based on the last MRI, the destruction of the knee joint was more prominent compare with the previous MRI, so it suggested the flexion limitation in this patient, which is more likely to be caused by the muscle stiffness. Because of the pain exacerbated during the movements of the patient’s joint, he tends to decrease the use of his knee joint, resulting in the muscle stiffness of the knee joint, which can be alleviated by regular physiotherapy.

Early secondary osteoarthritis is a common complication following knee tuberculosis. The patient must monitor, including regularly physiotherapy, that would give better outcomes for the patient himself. Therefore, continuous medical monitoring is needed annually for assessing the long-term complications of knee tuberculosis.

## 4 Conclusion

Knee tuberculosis is a rare disease that often misdiagnosed as bacterial infections or malignancy. Mimicking clinical manifestations needs further surgical approach followed by histopathology examination to diagnose this disease. Early recognition and prompt treatment were crucially required to avoid the permanent limitation of movement that could affect the patient’s quality of life. In this case, a lack of knowledge of the patient and his family significantly contributes to the delayed diagnosis.

## Declaration of competing interest

The authors have no financial or personal circumstances with pharmacists or organizations that could influence the originality of this manuscript.

## Source of funding

There is no financial funding that contributes to collecting, interpreting data, writing, and publishing the manuscript.

## Ethical approval

This study has been approved by Ethical committee of Faculty of Medicine Universitas Sumatera Utara 2020.

## Consent

Patients had obtained written consent for presenting and publishing of this case report, including the picture. The informed consent may be copied for the chief editor on request. There is no ethical issue of all identifying identities from this manuscript.

## Author contribution

Noni Novisari Soeroso and Heru Rahmadhany are the doctor in charge of this patients and have made substansial contribution of collecting, analysis and contributing data. Fannie Rizki Ananda is in charge of drafting and writing the article. Dedy Dwi Putra contributes as professional radiologist who interprets the radiological finding in this case. All authors have approved the final version of this manuscript before submitting into this journal.

## Registration of research studies

This article is a case report. No trials or new experiment was conducted in this study.

## Guarantor

The guarantor for this manuscript will be Dr. Noni Novisari Soeroso, the corresponding author.

## Provenance and peer review

Not commissioned, externally peer-reviewed.

## References

[1] Kulchavenya E. (2014). Extra-pulmonary tuberculosis: Are statistical reports accurate?. Ther. Adv. Infect. Dis..

[2] WHO (2019). Global Tuberculosis Report 2018.

[3] Pang Y., An J., Shu W. (2019). Epidemiology of extra-pulmonary tuberculosis among inpatients, China, 2008-2017. Emerg. Infect. Dis.

[4] Peto H.M., Pratt R.H., Harrington T.A., LoBue P.A., Armstrong L.R. (2009). Epidemiology of extra-pulmonary tuberculosis in the United States, 1993–2006. Clin. Infect. Dis..

[5] Lidder S., Lang K., Haroon M., Shahidi M., El-Guindi M. (2009). Tuberculosis of the knee. Orthop. Rev. (Pavia).

[6] Agha R.A., Borrelli M.R., Farwana R., Koshy K., Fowler A., Orgill D.P., For the SCARE group (2018). The SCARE 2018 statement: updating consensus surgical CAse REport (SCARE) guidelines. Int. J. Surg..

[7] Soeroso N.N., Harina E.G., Yosi A. (2019). A very rare case of scrofuloderma with multiple cervical lymphadenitis tuberculosis. Respir. Med. Case Reports.

[8] Soeroso Nn, Pradana A., Lubis N., Soeroso L. (2018). Successful treatment of total paraplegic patient due to tuberculous spondylitis. Respirol. Case. Reports.

[9] Paton R.Y. (1943). Tuberculosis of the knee joint. Postgrad. Med. J..

[10] Kerri O., Martini M. (1985). Tuberculosis of the knee. Int. Orthop..

[11] Hopewell P.C. (2019). TB Care 1. http://www.tbcare1.org/publicationshttp://www.istcweb.orghttp://www.currytbcenter.ucsf.edu/internationalhttp://www.who.int/tb/publications.

[12] Zamani B., Shayestehpour M. (2019). A case of knee monoarthritis caused by Mycobacterium tuberculosis. Am. J. Case Rep..

[13] Kumar S.N., Prasad T.S., Narayan P.A., Muruganandhan J. (2013). Granuloma with langhans giant cells: an overview. J. Oral Maxillofac. Pathol..

[14] Vyravan P.R., Choudhary B.M., Kumar M.M. (2014). http://www.iosrjournals.orgwww.iosrjournals.org.

[15] Sultan A.A., Cantrell W.A., Rose E. (2018). Total knee arthroplasty in the face of a previous tuberculosis infection of the knee: what do we know in 2018?. Expert Rev. Med. Devices.

[16] Zeng M., Xie J., Wang L., Hu Y. (2016). Total knee arthroplasty in advanced tuberculous arthritis of the knee. Int. Orthop..

[17] Su J.Y., Huang T.L., Lin S.Y. (1996). Total knee arthroplasty in tuberculous arthritis. Clinical Orthopaedics and Related Research.

[18] Misgar M.S., Mir N.A., Narbu T. (2019). Partial synovectomy in the treatment of tuberculosis of the knee. Int. Surg..

[19] Shen H.L., Xia Y., Li P., Wang J., Han H. (2010). Arthroscopic operations in knee joint with early-stage tuberculosis. Arch Orthop Trauma Surg.

[20] (2019). International Standard of TB Care 3. https://www.who.int/tb/publications/ISTC_3rdEd.pdf.

